# Supplementary material for: Intermittent preventive treatment with Sulphadoxine-Pyrimethamine (IPTp-SP) is associated with protection against sub-microscopic P. falciparum infection in pregnant women during the low transmission dry season in southwestern Cameroon: A Semi - longitudinal study
Source: PLoS One. 2022 Sep 30;17(9):e0275370. doi: 10.1371/journal.pone.0275370 (PMC9524640; doi:10.1371/journal.pone.0275370)
Supplement: Attachment — Submitted filename: Dear authors.pdf [file pone.0275370.s001.pdf]

Dear authors.

First, I want to congratulate your study. However, after analysis, some questions must be answered or justified before publication.

Their study deals with "Intermittent preventive treatment with Sulphadoxine-Pyrimethamine (IPTp-SP) is associated with protection against sub-microscopic P. falciparum infection in pregnant women during the low transmission dry season in southwestern Cameroon".

I. Abstract section: includes all necessary information. **II. Introduction:** It includes all the necessary information, so you could mention other studies that were effective in this context and even the limitation in your state of the art. This way we will have more robustness in the study. **III: Methodology:** where was the material used for detection, on filter paper, for example, or was the material in natura? – this information must be described; in addition, I did not identify the process of sample feasibility, before carrying out the PCR process; as well, the criteria for admission of the drug to the patient, dosage and/or conflicting factors of the process were not described. However, it would be ideal to demonstrate the adverse reactions that these patients had after using the drug. **IV: Results:** includes all necessary information. **V: Discussion:** would you be able to correlate this process with the possible pharmacogenomic influence on patients, perhaps, this will give you the necessary answers from both positive and negative results. Finally, I congratulate you on your study and wish you success.
