# Supplementary material for: Intermittent preventive treatment with Sulphadoxine-Pyrimethamine (IPTp-SP) is associated with protection against sub-microscopic P. falciparum infection in pregnant women during the low transmission dry season in southwestern Cameroon: A Semi - longitudinal study
Source: PLoS One. 2022 Sep 30;17(9):e0275370. doi: 10.1371/journal.pone.0275370 (PMC9524640; doi:10.1371/journal.pone.0275370)
Supplement: Attachment — Submitted filename: Comments-KK.docx [file pone.0275370.s002.docx]

**Comments:**

1. **Abstract:**

Line 39: A sentence should not start with number.

No perspectives were drawn from this study in the conclusion section.

Line 53: May be country to be added here.

2. **Introduction:**

Line 85: Harrington et al. 2012. There is a lack of consistency in the reporting of reference, as some are in number. Same for reference in line 95, ect….

Line 96: the term effect is not adequate for this type of study. Association is preferable, as indicated in the title of the paper.

3. **Method section:**

In the ethic section, the suggestion is to provide the reference number of the ethical approval.

Line 121: reference 16 is cited. In fact this sentence is not from that reference when assessed in the reference list.

Line 144: reference to be numbered.

Line 157: when was haemoglobin assessed? At enrolment or at delivery?

**Data analysis:**

The analytic section is less developed than it should be. The authors should provide more details here.

Also,

Line 200: what were the criteria of having a variable in the multivariate model in tables that content adjustment? Also, it is not clear in this section that logistic regression has been used, although OR was seen in the tables.

Line 202: Chi Square test was used. Can the authors provide the Chi Square test used?

As this is cohort study, what is the rationale of using odds ratio instead of risk ratio?

4. **Results:**

In table 2: Fever at enrolment; What is the difference between the Febrile and Axillar temperature >= 37.5?

The prevalence of low birthweight seems to be very low. What is the authors’ impression on this, as in many study conducted in sub Saharan Africa this is about 10% ?

Line 252: the numerator reported here (35) is different from the sample size (57) to estimate the geometric mean of parasitemia. Can the authors explain that?

Line 255: The term chemoprophylaxis is used. However, the authors were assessing IPTp. These two terms are different. The term IPTp is preferred. Same for line 289.

Line 303: the term birth weight is used in two words. However, in many parts of the document, it is written in a single word.

Line 307: Similarly, to line 260, the term Effect is not appropriate, as this is an association study and not effect study.

Table 4: The authors seem to have no difference in term of birth weight in different groups of IPTp. Can the authors provide any explanation of that?

Table 4 and Figure 3: There seems to be a discrepancy between Table 4 and Figure 3 regarding the association between IPTp usage and dosage and malaria infection. Is there any explanation of that?

5. **Discussion:**

There is a mixture of Harvard and Vancouver across all the discussion section. Please correct.

6. **Conclusion:**

One would like to have at least a sentence for perspective in the context of this study. Can the authors provide one?
