# Supplementary material for: Intermittent preventive treatment with Sulphadoxine-Pyrimethamine (IPTp-SP) is associated with protection against sub-microscopic P. falciparum infection in pregnant women during the low transmission dry season in southwestern Cameroon: A Semi - longitudinal study
Source: PLoS One. 2022 Sep 30;17(9):e0275370. doi: 10.1371/journal.pone.0275370 (PMC9524640; doi:10.1371/journal.pone.0275370)
Supplement: Attachment — Submitted filename: Response to Reviewer Comments_MIP Manuscript 09082022.docx [file pone.0275370.s003.docx]

| **Reviewer Comment** | **Response** |
| --- | --- |
| **Reviewer 2** |  |
| **Abstract:** |  |
| Line 39: A sentence should not start with number. | Corrected to start with ”About” 25% |
| No perspectives were drawn from this study in the conclusion section. | Future studies should be carried out in hyperendemic malaria foci where the parasitemia levels are substantially higher in order to confirm the efficacy of **IPTp-SP** |
| Line 53: May be country to be added here. | The country “Cameroon” where this study was undertaken has been added to the keywords as suggested |
| **Introduction:** |  |
| Line 85: Harrington et al. 2012. There is a lack of consistency in the reporting of reference, as some are in number. Same for reference in line 95, ect…. | All inconsistencies in citation and references have been corrected in line with journal specifications |
| Line 96: the term effect is not adequate for this type of study. Association is preferable, as indicated in the title of the paper. | This has been corrected in the manuscript to “relationship” since pregnancy outcomes were not associated submicroscopic infection |
| **Method section:** |  |
| In the ethic section, the suggestion is to provide the reference number of the ethical approval. | The reference number of the ethical approval (ref. N^o^2019/09/1188/CE/CNERSH/SP) has now been provide as suggested |
| Line 121: reference 16 is cited. In fact this sentence is not from that reference when assessed in the reference list. | Thanks for the correction and apologies for the mix-up. The correct reference (16 and not 15) has now been cited in the text. |
| Line 144: reference to be numbered. | Corrected as suggested |
| Line 157: when was haemoglobin assessed? At enrolment or at delivery? | Haemoglobin levels were assessed at enrolment and has now been indicated as such in the manuscript |
| **Data analysis:** |  |
| The analytic section is less developed than it should be. The authors should provide more details here. | This section has now been further developed and relevant details provided as recommended |
| Line 200: what were the criteria of having a variable in the multivariate model in tables that content adjustment? Also, it is not clear in this section that logistic regression has been used, although OR was seen in the tables. | The use of logistic regression and criteria for including variables in multivariate models has now been detailed in the analysis section |
| Line 202: Chi Square test was used. Can the authors provide the Chi Square test used?  As this is cohort study, what is the rationale of using odds ratio instead of risk ratio? | The corresponding chisquare statistics has now been incorporated into the table as suggested. The analysis in question relates to the risk of the participants at one time point (enrolment) and not following the semi-longitudinal outcome |
| **Results:** |  |
| In table 2: Fever at enrolment; What is the difference between the Febrile and Axillar temperature >= 37.5? | Fever at enrolment was classified in different ways. Current fever was defined as body temperature ≥37.5°C, with febrile individuals defined as participants with current fever or history of fever at enrolment. This has now been clarified under the “Data Collection and Definition of terms’ section in the manuscript |
| The prevalence of low birthweight seems to be very low. What is the authors’ impression on this, as in many study conducted in sub Saharan Africa this is about 10%? | This may be due to multiple factors including the status, nutrition and adherence of the mothers to control and intervention strategies. We note that most of the women were aged > 25 years, multigravidae, IPTp-SP users of atleast 3 doses at term, which factors have been shown to contribute to improved pregnancy outcome. |
| Line 252: the numerator reported here (35) is different from the sample size (57) to estimate the geometric mean of parasitemia. Can the authors explain that? | This can be explained by the fact that a few samples assessed for P. falciparum infection by peripheral blood smear microscopy were not assessed by PCR and vice versa. Such discrepancy would accrue to damaged slides during transportation/processing or indeterminate PCR results. |
| Line 255: The term chemoprophylaxis is used. However, the authors were assessing IPTp. These two terms are different. The term IPTp is preferred. Same for line 289. | This has been corrected in the manuscript as suggested |
| Line 303: the term birth weight is used in two words. However, in many parts of the document, it is written in a single word. | This has been corrected to a single word in the manuscript as suggested |
| Line 307: Similarly, to line 260, the term Effect is not appropriate, as this is an association study and not effect study. | This has been corrected in the manuscript as suggested |
| Table 4: The authors seem to have no difference in term of birth weight in different groups of IPTp. Can the authors provide any explanation of that? | Indeed. Although there was a trend in creasing birthweight with IPTp-SP use, there was no difference in different IPTp-SP groups. This suggest that although the chemotherapy is associated with protection from sub-microscopic parasitaemia, this does not translate into pregnancy outcome in terms of birthweight. It is possible that the low numbers of non IPTp-SP users at term may not be statistically powered to detect difference, if they do exist. |
| Table 4 and Figure 3: There seems to be a discrepancy between Table 4 and Figure 3 regarding the association between IPTp usage and dosage and malaria infection. Is there any explanation of that? | Table 4 and Figure 3 present completely different information vis-à-vis IPTp usage and dosage.  While Table 4 compares pregnancy outcome (birthweight and APGAR score) across different IPTp usage and dosage groups, Figure 3 shows the association between IPTp-SP usage and dosage and the malaria parasite infection status |
| **Discussion:** |  |
| There is a mixture of Harvard and Vancouver across all the discussion section. Please correct. | All inconsistencies in citation and references have been corrected in line with journal specifications |
| **Conclusion:** |  |
| One would like to have at least a sentence for perspective in the context of this study. Can the authors provide one? | To conduct similar studies in a hyperendemic setting with higher parasitaemia levels and interrogate if IPTp induces protective responses to malaria |

| **Reviewer Comment** | **Response** |
| --- | --- |
| **Reviewer 1** |  |
| **I. Abstract section** |  |
| includes all necessary information | All necessary information has been included |
| **II. Introduction** |  |
| It includes all the necessary information, so you could mention other studies that were effective in this context and even the limitation in your state of the art. This way we will have more robustness in the study. | All necessary information has been included |
| **III: Methodology:** |  |
| where was the material used for detection, on filter paper, for example, or was the material in natura? – this information must be described; in addition, I did not identify the process of sample feasibility, before carrying out the PCR process; as well. | This has been corrected in the manuscript to include details on filter paper type and sample storage conditions prior to PCR testing. The study describes status in natura as testing was done on samples directly collected from the women enrolled. |
| the criteria for admission of the drug to the patient, dosage and/or conflicting factors of the process were not described. However, it would be ideal to demonstrate the adverse reactions that these patients had after using the drug. | A subsection detailing IPTp-SP usage and dosage information has now been included in the methodology section. Note that the previous drug use was self-reported or documented from medical records and subsequent use captured by directly observed administration by health care personnel. |
| **IV: Results:** |  |
| includes all necessary information. | All necessary information has been included |
| **V: Discussion** |  |
| would you be able to correlate this process with the possible pharmacogenomic influence on patients, perhaps, this will give you the necessary answers from both positive and negative results. Finally, I congratulate you on your study and wish you success. | It would be interesting to correlate out observation with pharmacogenomics influence on participants, a perspective in view of future studies. Thank you for the brilliant suggestion. |
